# Establishment of a Standardized Liver Fibrosis Model with Different Pathological Stages in Rats

**DOI:** 10.1155/2012/560345

**Published:** 2012-06-12

**Authors:** Li Li, Zongqiang Hu, Wen Li, Mingdao Hu, Jianghua Ran, Peng Chen, Qiangming Sun

**Affiliations:** ^1^Ganmei Hospital, Kunming Medical University, Kunming 650000, China; ^2^The Second Affiliated Hospital, Kunming Medical University, Kunming 650000, China; ^3^Molecular Epidemiology Joint Laboratory, Institute of Medical Biology, Kunming 650000, China

## Abstract

*Objective*. To establish a standardized animal model for liver fibrosis with the same assessment criteria for liver fibrosis studies that have been established on a unified platform. *Methods*. The standardized liver fibrosis model was established using Sprague-Dawley (SD) rats that either received an intraperitoneal injection of carbon tetrachloride (CCl_4_) in small dosages or ingested an ethanol solution. *Results*. The definite corresponding rules among modeling of different weeks and corresponding serology indices as well as different pathological staging can be observed by modeling with small dosages and slow, individualized, and combined administrations. *Conclusion*. This method can be used for the standardized establishment of a liver fibrosis model in rats across 5 pathological stages, ranging from S0 to S4, with a high success rate (89.33%) and low death rate (17.3%) because of the application of multiple hypotoxic chemicals for modeling. We refer to the criteria of Histological Grading and Staging of Chronic Hepatitis for Fibrosis established by the 10th World Digestive Disease Academic Conference in Los Angeles in September 1994 (revised in November 2000).

## 1. Introduction

Currently, there are hundreds of million patients with liver fibrosis throughout the world, and most cases are caused by hepatitis viral infections [1–7]. In Asian countries, the infection rate of hepatitis B has reached 10% of the total population [8–10], whereas hepatitis C virus (HCV) infection rates in the western countries remain high. Dr. Hans Popper, an authority on liver diseases in the USA during the 20th century, has pointed out that “anyone who can stop or delay liver fibrosis would be able to cure most chronic liver diseases.” Viral hepatitis, posthepatitic cirrhosis, and hepatoma are considered to be the three steps of liver disease. Most studies on liver diseases throughout the world are mainly focused on the interruption of pathological progression to prevent or delay the genesis of hepatic cirrhosis and hepatoma.

Hepatic cirrhosis is a progressive consequence of liver fibrosis. Liver fibrosis refers to the accumulation of extracellular matrix (ECM) proteins, which occurs in most types of chronic diseases. Of these proteins, collagen, which is the body's self-repairing protein induced by inflammation, most commonly accumulates. The formation and progression of liver fibrosis is an extremely complicated, gradual process, which is mainly reflected as sustainable inflammation in the liver inducing an ecological disequilibrium of cells and intracellular substances. Therefore, antiliver fibrosis therapy is a combined therapy targeting the cause of liver fibrosis and delaying and interrupting the pathological progression. Only in this way can we control the progression of chronic hepatitis B infection into liver fibrosis and hepatic cirrhosis.

Currently, increasingly more studies are focused on liver fibrosis using animal models. However, the unified assessment of these studies' results is difficult due to multiple modeling methods and different qualities of modeling. Therefore, it is difficult to generalize some achievements that seem to have significance because of the lack of an effective assessment of a standardized animal model. The establishment of a standardized animal model for liver fibrosis showed critical significance in the study of new clinical therapies, optimal therapeutic opportunities, assessment of therapeutic effects, academic communications, and so forth. 

Recently, more intensive studies within the pathology community have been performed on liver fibrosis, and the definite diagnostic criteria of liver fibrosis in stages 0–4 have been defined [11], which provided a significant reference for animal studies of liver fibrosis and a higher respect for the investigators [12–14]. Undoubtedly, the criteria play critical roles in the selection of optimal therapeutic opportunities, assessment of therapies to reverse liver fibrosis, and in basic research. However, there are few reports on standardizing the modeling of the different pathological stages of liver fibrosis throughout the world. Therefore, we simulated the natural pathological process of liver fibrosis in male Sprague-Dawley (SD) rats as accurately as possible using hypotoxic drugs with combined, low-dose, individualized, and long-term administration to simulate the pathological changes of injury. Meanwhile, we found a definite method to establish a pathological staging model of liver fibrosis, which pave a way for the assessment of therapeutic effects in certain pathological stages.

## 2. Materials and Methods

### 2.1. Laboratory Animals

A total of 95 male Sprague-Dawley (SD) rats weighing 280 g to 300 g were provided by the Animal Department of Kunming Medical College. The rats were housed in separate cages under a 12-hour light/dark cycle with access to food and water ad libitum. The room temperature was kept around 25°C, and humidity was 60–70%. The rats were anesthetized with ether and sacrificed for histopathologic examination following euthanasia.

The welfare of the animals in this study was based on the regulations of *Good Laboratory Practice* issued in 1978 by the US Food and Drug Administration (FDA) and *Drug Good Laboratory Practices (Transitional)* issued by the State Food and Drug Administration (SFDA) in China.

#### 2.1.1. Reagents

The following reagents were used in this study: 5% ethanol solution, analytical reagent carbon tetrachloride (CCl_4_), edible olive oil, analytical reagent ethyl ether, and 10% neutral formaldehyde buffer (4 g KH_2_PO_4_ and 6.5 g Na_2_HPO_4_ dissolved in 900 mL distilled water, and formaldehyde was added to a total volume of 1000 mL).

#### 2.1.2. Methods

The 10% CCl_4_ liver oil solution was prepared with analytical reagent CCl_4_ and olive oil at a volume ratio of 1 : 9. The 5% ethanol was prepared with dehydrated ethanol solution and distilled water at a dilution of 1 : 20. The rats were raised for 1 week without adverse reactions. The rats with normal water, food, and activity were collected in the study. A total of 95 rats were randomized into 2 groups with 20 members in the control group receiving an intraperitoneal injection of normal saline and 75 members in the animal model group using intraperitoneal and subcutaneous injections of 10% CCl_4_ liver oil solution at a dose of 1 mL/kg administered twice a week for 15 weeks. The 5% edible ethanol solution was used as the only drink for experimental rats. Five rats in the control group were sacrificed each week from the 2nd to the 15th week, and the blood-related indices were detected for hepatic pathological analysis. 

#### 2.1.3. Outcomes

The following general conditions of rats (including death, activity, appetite, weight, appearance, and feces) were observed.

#### 2.1.4. Serology Indices

The serum hyaluronic acid (HA), laminin (LN), precollagen III N-terminal (PIIINP), and type IV collagen (CIV) levels were detected using radioimmunoassay. The connective tissue growth factors (CTGFs) were detected by enzyme-linked immunosorbent assay (ELISA), according to manufacturer's instructions.

#### 2.1.5. Hepatic Pathology of Rats

The size, shape, texture, and color of diseased livers were obtained from live tissue at the same location, fixed by 10% neutral-balanced formalin solution, and 4 *μ*m serial sections were obtained, followed by paraffin imbedding. Subsequently, HE (Hematoxylin and Eosin) and VG (Van-Gieson) staining was used to observe the structural changes of hepatic tissue and hyperplasia of collagen fibers which can be observed in pathological sections. The pathological grading was based on the criteria of Histological Grading and Staging of Chronic Hepatitis for Fibrosis established by the 10th World Digestive Disease Academic Conference in Los Angeles in September 1994. (revised in November 2000) [11, 15]: S0 stage, no fibrosis; S1 stage, expansion of fibrosis in portal area, localized perisinusoidal and intralobular fibrosis; S2 stage, peripheral fibrosis in portal area, formation of fibrous septum, retention of intralobular architecture; S3 stage, fibrous septum accompanied by intralobular structural disorders, no hepatic cirrhosis; S4 stage, early hepatic cirrhosis. The liver tissue was prepared with conventional sectioning and HE staining, and fibrosis was observed under light microscopy [16–18].

### 2.2. Statistical Analysis

All data are expressed as the mean ± SD. The SPSS 16.0 software (SPSS Inc., Chicago, IL) was used for statistical analysis. Student's *t*-test was used for the comparison between the means of two samples. ANOVA was applied for the comparison among the means of multiple samples. 

## 3. Results

### 3.1. The Success Rate of Liver Fibrosis Model

The modeling of liver fibroses was successful in 67 cases in 75 rats, and 13 cases died. The modeling success rate was 89.33%, and the death rate was 17.3% (consisting of 1 dead case in the 6th week, 2 dead cases in the 8th week, 1 dead case in the 9th week, 1 dead case in the 11th week, 2 dead cases in the 12th week, 1 dead case in the 13th week, 1 dead case in the 14th week, 1 dead case in the 15th week, and 2 dead cases in the 16th week). The formation of fibrosis in rats in the modeling group gradually showed the characteristic manifestations of the different pathological stages from S0 to S4 as the weeks elapsed. The diffuse suppurative inflammation was observed in the abdominal cavity of dead rats during modeling at autopsy, and necrosis in an extensively large area of liver tissue accompanied by neutrophil infiltration was observed in liver tissue upon histological observation.

### 3.2. General Status of Rats

**Figure 1:**
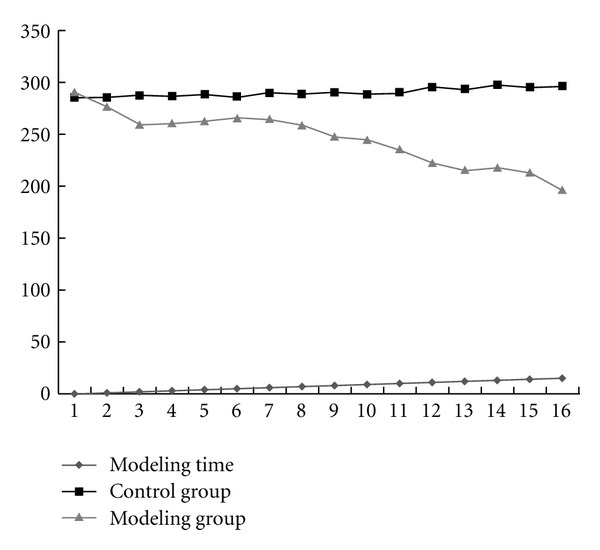
Comparison of weight changes of rats between the groups in each week.

The general status of the rats in the control group was excellent, and the weight of rats increased rapidly. The rats in the modeling group showed weak glossiness of pelage, depressed spirit, depressed appetite, and a total death rate of 10.67%. The weight changes in the two groups, as a function of modeling time, are shown in Figure 1.

### 3.3. Detections of Serology

The levels of serum HA, LN, PIIINP, CIV, and CTGFs in the modeling group were gradually enhanced from the 1st week. The comparison of these indices in the modeling groups among different weeks and comparison between the modeling group and control group in same week showed a significant difference, whereas the serum HA, LN, PIIINP, and CIV levels in the control group showed no significant difference in any week (Figure 1). The serum CTGF levels in the modeling group were significantly higher than in the control group (Table 1).

### 3.4. Hepatic Pathology in Rats

**Figure 2:**
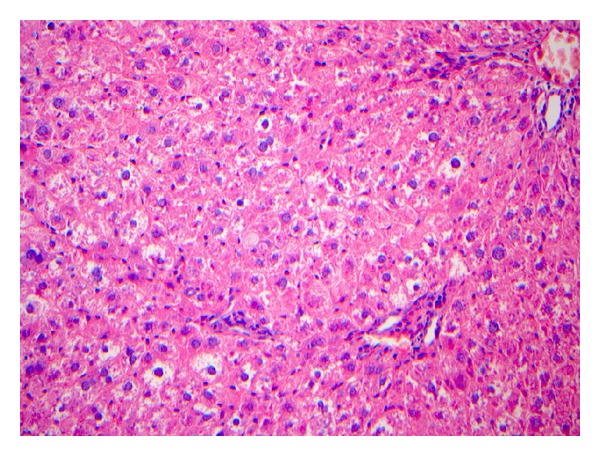
The pathological stage of the 2nd–4th week is S1. The figure describes the general appearance of liver pathology: the liver was slightly swollen, the color was slightly gloomy, and the texture was normal. Under the microscope, the hepatic lobular architecture was integrated, the structure of chloasma hepaticum was not clear, the hepatocytes showed vacuolar degeneration, the structure of the portal area was still clear, and no fibrous hyperplasia was observed. HE. Bar 5200 mm.

**Figure 3:**
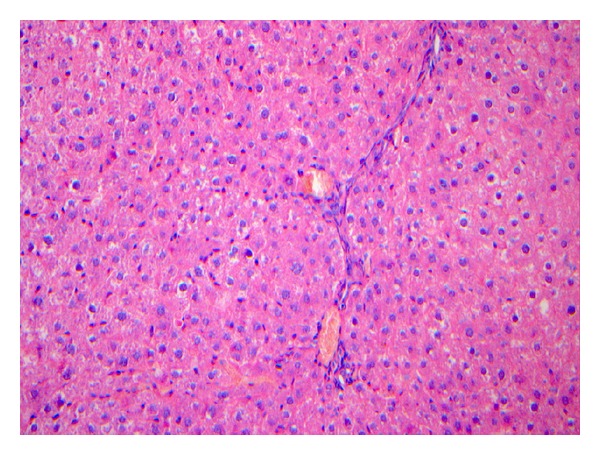
The pathological stage of the 5th–8th week is S2. The figure was the general appearance of liver pathology: there were evident swellings of the liver. Under the microscope, the hepatic lobular architecture was unclear, the arrangement of hepatic plate was disorganized, the hepatocytes showed vacuolar degeneration, and the hepatocytes showed necrosis in fragments. The liver sinusoid was narrowing or disappeared by compression. The fibrosis could be observed in the portal area but was localized in the lobule. HE. Bar 5200 mm.

**Figure 4:**
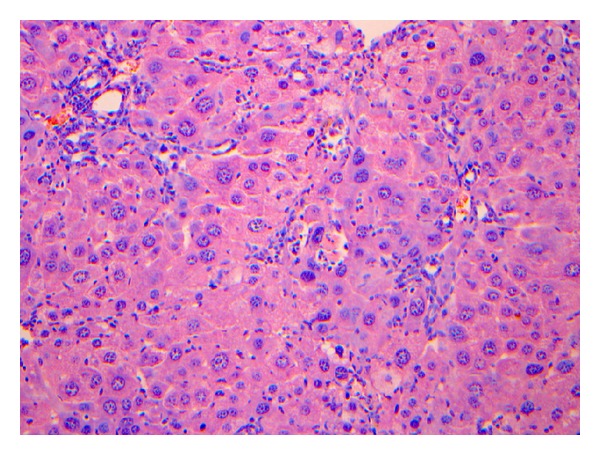
The pathological stage of the 9th–12th week is S3. The figure was the general appearance of liver pathology under the microscope: the aforementioned conditions of live rats were aggravated, the peripheral fibrosis in the portal area could be observed, and the formation of fibrous septum could be observed locally. HE. Bar 5200 mm.

**Figure 5:**
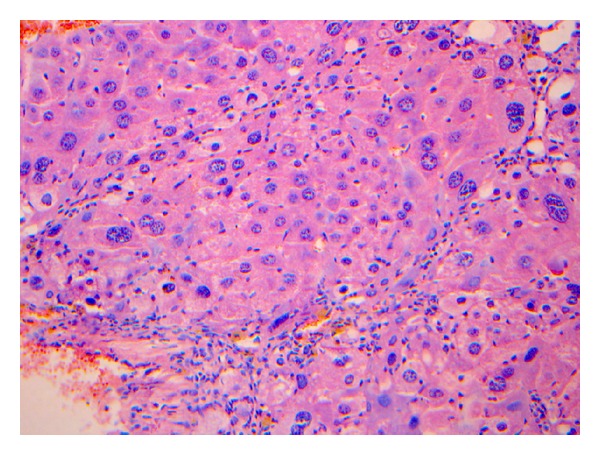
The pathological stage of the 13th–14th week is S4. The figure was the general appearance of liver pathology under the microscope: fibroblasts in the portal area showed evident hyperplasia, the fibrous septum was widened, the grid division was formed in liver cells, the pseudo-lobule of the liver was formed, and a typical liver fibrosis was formed, which indicated it was at the stage of hepatic cirrhosis. HE. Bar 5200 mm.

The liver pathology of rats in the modeling group were reflected by the characteristics shown in Figures 2–5.

## 4. Discussion

### 4.1. The Quality Control of the Establishment of a Standardized Liver Fibrosis Model

The modeling methods of liver fibrosis include the induction of a hepatotoxicant, induction of a high fat diet, immunization, bile duct obstruction, parasite methods, dystrophy methods, and a combined application of the aforementioned methods [19, 20]. CCl_4_ was used as an experimental material for hepatic injury, according to the principle of chronic hepatic and kidney impairment induced by low-dosage and multiple administrations of CCl_4_ since the 1930s. Until recently, CCl_4_ was still the most extensively used hepatic-toxic substance for the induction of liver fibrosis in rats [21, 22]. The specific mechanism might have taken the following course: CCl_4_ entered into the animal hepatic tissue, produced trichloromethyl and trichloromethyl peroxide by oxidation in the endoplasmic reticulum of hepatic cells via a cytochrome P450-dependent mixed-function oxidase. These free radicals switched on the unsaturated lipid membrane peroxidation, induced serious destruction of the hepatic cell membrane and structure of organelles, and caused hepatic cell injury, degeneration, necrosis, and liver fibrosis formation induced by a long-term repeated stimulus [23–25].

The hepatic cellular impairment can be induced 15 min after CCl_4_ enters the body, which achieved the peak value at 48 h. Subsequently, the liver entered the renovation stage, and the administration was performed with an interval of 3-4 d. Therefore, readministration during the renovation stage was performed after hepatic injury was caused by one toxic infection, the renovation was injured repeatedly, and liver fibrosis was induced. The animal model of liver fibrosis induced by CCl_4_ showed morphologic and pathophysiologic aspects similar to human liver fibrosis. Moreover, the breeding of rats was simple, the modeling in rats was easy, the time cost was low, and the pathological features were stable and reliable [26, 27]. The animal modeling of liver fibrosis using CCl_4_ includes intragastric administration via the mouth, intraperitoneal injection, and subcutaneous injection [28–30]. The following were the characteristics of intraperitoneal and subcutaneous injections. (1) The degree of liver fibrosis is ideal, and liver fibrosis after hepatitis B infection could be simulated by the pathological appearance. (2) The progression of liver fibrosis was directly correlated with the time elapsed since injection, which is easy to control for the degree of fibrosis and beneficial for further studies. (3) Once the operative technique is mastered, the modeling is easy to perform, and the death rate of rats is relatively low. As it is easy to achieve a relatively low death rate of modeling with this technique, the aforementioned methods are becoming the most commonly administered methods.

In preliminary experiments, we observed that rats showed significant differences after injection of CCl_4_ due to varying animal weights, ages, dosage, and time interval between two administrations; for instance, small weight, older age, high dose, and low-time interval between administrations might induce acute poisoning and death. We considered the following differences. (1) The weight of modeling rats should be 250 g–300 g, and rats with a lower weight are more prone to die, whereas the rats with a higher weight are challenging in standardized modeling because of differences in hepatic detoxication. (2) The focus should be placed on the approach of injection, and sufficient experiences should be accumulated. If the drug solution enters the intestinal canal, the rats must die of acute stomach (intestinal) toxic expansion. If the drug solution enters the blood vessels, the rats must die of acute poisoning. To attenuate the effects of the struggle of rats during the operation and to avoid biting, we used plastic bottles with the bottoms removed. The head and body from the umbilicus of the rat were secured in the bottle, and one hind leg of the rat was fixed. Subsequently, the prepared drug solution was injected into the abdominal cavity of the hypogastric zone; therefore, the rat cannot hurt the human, and it is easy to fix the position of injection. (3) The sensations of “breakthrough” and “liberation” from needle tip needed to be identified after the injection of the solution into the abdominal cavity. The administration was complete when the solution was injected into the abdominal cavity. (4) The environmental temperature, humidity, noise, and light should be controlled during the procedures of animal modeling, and these factors might affect the amount of fluid intake, which is not beneficial for the standardization of the animal model. 

Based on the liver fibrosis model, multiple mechanisms are involved in the progression of liver fibrosis pathology. We tried to control potential factors, including weight, age (in weeks), and environment, we used CCl_4_ with ethanol solution for modeling, and we explored the feasibility and effectiveness of these models for standardization [12, 31, 32]. Meanwhile, we observed the correlations between serology indices of liver fibrosis, including HA, LN, PIIINP, CIV, CTGFs, and pathological staging. We also confirmed the feasibility of the establishment of a standardized animal model for liver fibrosis [33, 34]. Moreover, we developed a substantial foundation for the establishment of a serology-based, nontrauma model of liver fibrosis and standardized studies in the clinical setting.

### 4.2. Critical Significance of Establishment of a Standardized Liver Fibrosis Model in Rats in Different Pathological Stages

It is confirmed that we have found a good deal of ways to treat liver fibrosis. However, we have no unified standard to evaluate their effectiveness. The establishment of a standardized animal model in different pathological stages might pave the way for opportunities in the biological therapy of liver fibrosis and provide a standard for the assessment of therapeutic effects.

## Figures and Tables

**Table 1:** Results of related indices in different stages (mean ± SD).

Stages of hepatic fibrosis	*n*	HA	LN	PIIIP	CIV	CTGF
S_0_	10	66.59 ± 6.89	107.81 ± 12.04	86.75 ± 19.26	58.54 ± 14.12	0.20 ± 0.13
S_1_	11	106.29 ± 19.49	110.60 ± 14.43	105.68 ± 20.03	66.95 ± 17.06	0.41 ± 0.10
S_2_	22	164.46 ± 37.09	117.24 ± 19.86	114.04 ± 19.24	90.99 ± 27.15	0.59 ± 0.07
S_3_	11	251.66 ± 74.75	139.95 ± 25.78	156.96 ± 27.18	103.79 ± 25.41	0.76 ± 0.09
S_4_	13	342.54 ± 80.72	132.26 ± 23.94	170.28 ± 17.91	137.83 ± 41.56	0.99 ± 0.03
F		54.52	5.431	34.753	15.320	149.680
P		0.000^∗^	0.001^∗^	0.000^∗^	0.000^∗^	0.000^∗^

*No* *te* *s*. ^∗^denotes *P* < 0.002, and the difference showed statistical significance.

Pairwise comparison. HA: pairwise comparison between S0 and S1 stage showed no significant difference, whereas pair comparisons of other stages showed a significant difference. LN: pairwise comparison between S0 and S1 stage, S0 and S2 stage, and S3 and S4 stage showed no significant difference, whereas pair comparisons of other stages showed statistical significance. PIIIP: pairwise comparison between S1 and S2 stage and S3 and S4 stage showed no significant difference, whereas pair comparisons of other stages showed statistical significance. CIV: pairwise comparison between S0 and S1 stage and S2 and S3 stage showed no significant difference, whereas pair comparisons of other stages showed statistical significance. CTGF: the comparison between different liver fibrosis stages showed statistical significance.
